# Diagnostic delay, treatment duration and outcomes since the implementation of integrated model of tuberculosis control and their associated factors in a county in East China

**DOI:** 10.1186/s12879-023-08561-w

**Published:** 2023-10-25

**Authors:** Yuanxiang Xie, Ting Ting Shum, Zhenming Tian, Chuanheng Lin, Lingyuan Chen, Bin Chen, Dajiang Huang, Lei Zhu, Guanyang Zou

**Affiliations:** 1 School of Public Health and Management, Guangzhou University of Chinese Medicine, Guangzhou, China; 2 Department of Social Anthropology, School of Social and Political Science, University of Edinburgh, Edinburgh, UK; 3 Center for Public Health, Longgang County, Wenzhou, Zhejiang Province China; 4 Center for Disease Prevention and Control, Cangnan County, Wenzhou, Zhejiang Province China; 5 Zhejiang Provincial Center for Disease Prevention and Control, Hangzhou, China; 6 School of Postgraduate Studies, Guangzhou University of Chinese Medicine, Guangzhou, China

**Keywords:** Tuberculosis control, Diagnostic delay, Treatment duration, Treatment outcome, Integrated model, China

## Abstract

**Objective:**

This study assesses the diagnostic delay, treatment duration and treatment outcomes of tuberculosis (TB) patients since the implementation of the integrated model of TB control in a county in eastern China. It further identifies factors associated with diagnostic delay and treatment duration in the integrated model.

**Methods:**

We collected data through the Chinese Tuberculosis Information Management System (TBIMS) for Cangnan County in Zhejiang Province. Chi-square and Mann-Whitney tests were adopted to identify factors associated with duration of treatment and treatment delay for TB patients within the integrated model. Multiple regression analysis was subsequently performed to confirm the identified factors.

**Results:**

In the integrated model from 2012 to 2018, the median health system delay was maintained at 1 day, and the median patient delay decreased from 14 to 9 days and the median total delay decreased from 15 to 11 days. In addition, the proportion of patients who experienced patient delay > 14 days and total delay > 28 days decreased from 49% to 35% and from 32% to 29% respectively. However, the proportion of patients who had health system delay > 14 days increased from 0.2% to 13% from 2012 to 2018. The median treatment duration increased from 199 to 366 days and the number of TB patients lost to follow-up showed an overall upward trend from 2012 to 2018. The multivariable regression analysis indicated that migrant TB patients and TB patients initially diagnosed in hospitals at the prefectural level and above tended to experience total delay > 28 days (p < 0.001). Linear regression analysis confirmed that new TB patients>60 years tended to have longer treatment duration (p < 0.05).

**Conclusions:**

While our study may suggest the potential of the integrated model in early detection and diagnosis of TB, it also suggests the importance of strengthening supervision and management of designated hospitals to optimize the treatment duration and improve retention of patients in TB care. Enhancing health education for TB patients, especially amongst migrant patients, and training in TB identification and referral for non-TB doctors are also key for early TB detection and diagnosis in the integrated model.

## Introduction

Tuberculosis (TB) is an airborne infectious disease [1]. It continues to proliferate as a significant global health problem, with around 10 million new TB cases and 1.4 million deaths in 2019 [1]. China has made great strides in tackling TB but still faces a considerable burden of disease [1]. In 2019, China reported 833,000 TB cases, making it the country with the second-highest TB burden in the world [1]. In China, there are two main models of TB management and treatment: the dispensary model and the integrated model. Both follow the World Health Organization (WHO) advocated Directly Observed Therapy Short-course (DOTs) approach for TB control [2] In the “dispensary model”, the TB dispensary is a department usually affiliated with the Center for Disease Prevention and Control (CDC) at the county level. The TB dispensary provides clinical and public health care, and general hospitals should refer presumptive TB cases to these TB dispensaries [3]. However, the clinical capacity and medical resources in TB dispensaries are believed to be poorer than those of the public hospitals and integration of TB clinical services into public hospitals is expected to reduce delays in diagnosis for TB patients and improve the quality of clinical care for TB [4, 5]. Following the implementation of the “12th Five-Year” plan in 2011, the “integrated model” has gradually replaced the “dispensary model” as the key model of TB control within most provinces in the country [6]. In the integrated model, a designated general public hospital– called “designated hospitals” – is authorized to diagnose and treat TB in the county. Medical personnel working at the village level, in the township level and in general hospitals within the county catchment area must refer TB patients or presumptive TB cases to the TB clinic of the designated hospital for diagnostic confirmation and treatment [3]. At the same time, the TB dispensaries of the CDC continue to provide public health functions such as case supervision, tracing, mass education, and training [3, 7].

In TB control, early diagnosis and timely treatment of tuberculosis are key to avoiding unfavorable outcomes [8]. Previous studies have shown that diagnostic delay and repeated clinical visits can influence the successful control of TB [9]. Furthermore, the integrated model is associated with shorter provider or health systems delays and fewer patient pathways compared with the dispensary model [4, 10]. And Wei et al. have reported that the integrated model has shorter patient delays and total delays as compared with the dispensary model [7]. However, other studies have shown that designated hospital healthcare providers in China have poor compliance with the national guidelines for TB treatment, which may be partly due to the perverse incentives of public hospitals as a result of underfunding [3]. Thus TB patients suffer from heavy financial burdens despite the free diagnosis and treatment policy [11], due to the prescription of non-recommended drugs, non-recommended examinations for TB treatment [12] and longer than recommended treatment duration [13].

To date, very few studies have included treatment duration in their analysis and a comprehensive understanding of the treatment processes of TB patients is lacking in the research of the integrated model. This study will assess the delayed diagnosis, treatment duration and treatment outcomes of TB patients since the implementation of the integrated model in a country in eastern China. It will further identify the factors in the integrated model causing these delays and the consequent prolongation of treatment duration that deviates from national guidelines.

## Methods

### Study design

This is a retrospective case study of the diagnostic delay, treatment duration and treatment outcomes of TB patients registered under the integrated model for TB control between 2012 and 2018. We use a dataset generated from the Chinese TB Information Management System (TBIMS, TB special reporting system version 2.0) for Cangnan County [14].

### Study setting

This study was carried out in Cangnan County, situated under the jurisdiction of Wenzhou City in Zhejiang province. Zhejiang is one of the most developed and populous provinces in China. Wenzhou is a municipality under the Provincial Government of Zhejiang Province. In 2018, the population of Wenzhou City was 9.3 million with a GDP of $9,800 per capita [15]. Cangnan County is one of five counties in Wenzhou City and has a population of 1.25 million and a GDP of $6,800 per capita [15]. Cangnan adopted the integrated model in 2011. Since that time, the diagnosis and treatment of TB patients in this county has been transferred from the CDC dispensary to the designated hospital. The CDC dispensary no longer provides diagnosis of and treatment of TB, but continues to provide public health functions, such as case supervision, tracing, mass education, and training for TB staff. Cangnan County People’s Hospital is the only designated hospital for TB in Cangnan, which provides diagnostic confirmation, treatment and case management of TB. Medical personnel in the non-TB clinic of the designated hospital, and those working in the village clinics, townships and other hospitals within the county must refer TB patients or presumptive TB cases to the TB clinic of the designated hospital. An infectious disease hosptial, which is also designated to provide TB care, is available at the prefectural level. Certain hospitals with diagnostic capacity, either within or outside the county, are eligible to provide TB diagnosis and may admit severe patients for treatment.

### Data collection

The Cangnan CDC of Zhejiang Province exported patient data between 2012 and 2018 from the TBIMS using Microsoft Excel (Microsoft, Redmond, WA, USA). The data provided information on the general characteristics of patients, detailed information related to instances of health services contact, and clinical information such as severity and cavity. This information was collected and recorded by the TB staff at the time of TB consultation and registration in the designated hospital. The county CDC conducted a quality check of this data. The integrated model was implemented in the second half of 2011. The year 2011 was perceived to be a transitional year from the dispensary model to the integrated model. We therefore did not include the 2011 data for analysis.

### Definitions

Definitions of different types of delays are described elsewhere [14, 16]. In this study, diagnosis delay covers patient delay, health system delay and total delay. Patient delay is defined as the time interval between symptom onset and first visit to a medical institution. Health system delay is defined as the time between the first visit to a medical institution and confirmation of TB diagnosis, mainly in the TB clinic of the designated hospital but also in other hospitals eligible to diagnose. Total delay is defined as the total sum of patient delay and health system delay [16]. Patient delay and health system delay was analysed using a dividing point of 14 days and total delay was analysed using a diving point of 28 days, as based on previous studies [17, 18]. Diagnosis of TB is mainly based on sputum smear examination, supplemented by sputum culture and X-Ray. To diagnose TB, presumptive patients must have three samples of smear sputum collected and tested; i.e., a sample of “instant sputum” taken in clinic in the designated hospital on the day of the first visit, a second sample of “night sputum” taken at home that evening, and a third sample of “morning sputum” taken at home the next day [19]. Finally, the time between a confirmed TB diagnosis and completed treatment is defined as treatment duration. According to the guidelines for implementing the National TB Control Program in China (version 2008), the standardized treatment courses for drug-susceptible TB in new and retreated patients are 6 and 8 months respectively [19]. The present study takes treatment outcome as classified into treatment success (completed treatment and cured status) and unfavorable outcome (failed treatment, died and lost) [19, 20].

### Data analysis

SPSS 25.0 (SPSS, Inc., Chicago, USA) was used for descriptive statistics and multiple regression analysis. Continuous variables were described by median and IQR, and categorical variables were calculated by counts and percentage. Univariate analysis, using the Chi-square test and Mann-Whitney U test, was adopted to identify the factors associated with delay and treatment duration for TB patients in the integrated model. Those with a *p* value < 0.2 in the Chi-square test and Mann-Whitney U Test were included in the subsequent linear regression and binary logistic regression, where appropriate, using a backward method. Adjusted ORs with 95% CI were presented with P < 0.05 being treated as statistically significant.

## Results

### General and clinical characteristics of TB patients from 2012 to 2018

In total, 3,756 TB cases were recorded between 2012 and 2018 in Cangnan County. The number of TB patients ranged between 440 and 650 each year from 2012 to 2018. From 2012 to 2018, the median age of TB patients ranged between 39 and 50; the proportion of patients who were above 60 years ranged between 19% and 31%; male patients ranged between 67% and 74%; migrant patients ranged between 9% and 24%; farmers ranged between 49% and 83%; smear positive patients ranged between 28% and 38%; new patients ranged between 86% and 88%; severe cases ranged between18% and 39%; patients with cavities ranged between 34% and 40%; and those who were initially diagnosed at county-level hospitals showed an downward trend, decreasing from 99.7 to 82% (Table 1).

**Table 1:** Socio-demographic and clinical characteristics of TB patients from 2012 to 2018

	2012	2013	2014	2015	2016	2017	2018	*P*-value
	n=(650)	n=(607)	n=(528)	n=(564)	n=(488)	n=(479)	n=(440)	
**Age (Median, IQR)**	41(26–57)	39(24–57)	45(30–58)	46(30–60)	45(28–58)	50(35–64)	46(29–60)	*P* < 0.001
**Age>60y (n, %)**	127(19)	122(20)	114(22)	133(24)	106(22)	149(31)	103(23)	*P* < 0.001
**Male (n, %)**	439(67)	446(74)	386(73)	405(72)	355(73)	350(73)	314(71)	*P* = 0.258
***Hukou *** **status (n, %)**								*P* < 0.001
Local	579(89)	554(91)	456(86)	477(85)	397(81)	382(80)	336(76)	
Migrant	71(11)	53(9)	72(14)	87(15)	91(19)	97(20)	104(24)	
**Farmer (n, %)**	382(60)	361(60)	371(70)	470(83)	380(78)	337(70)	217(49)	*P* < 0.001
**Smear sputum results (n, %)**								*P* = 0.022
Negative	433(67)	394(65)	352(67)	353(63)	350(72)	306(64)	271(62)	
Positive	217(33)	213(35)	176(33)	211(37)	138(28)	173(36)	169(38)	
**Treatment classification (n, %)**								*P* = 0.859
New patients	573(88)	529(87)	454(86)	498(88)	422(86)	413(86)	386(88)	
Retreated patient	77(12)	78(13)	74(14)	66(12)	66(14)	66(14)	54(12)	
**Severe cases** ^**a**^ **(n, %)**	119(18)	210(35)	170(32)	207(37)	178(37)	186(39)	134(31)	*P* < 0.001
**Cavity (n, %)**	218(34)	211(35)	185(35)	218(39)	184(38)	191(40)	156(36)	*P* = 0.263
**Hospital level of initial diagnosis for TB (n, %)**								*P* < 0.001
County	648(99.7)	591(97)	502(95)	524(93)	430(88)	416(87)	362(82)	
Prefectural and above	2(0.3)	16(3)	26(5)	40(7)	58(12)	63(13)	78(18)	

^a^TB patients with large cavities or lesion in more than two lung lobes

### Diagnostic delay, treatment duration and treatment outcomes of TB patients from 2012 to 2018

**Fig. 1:**
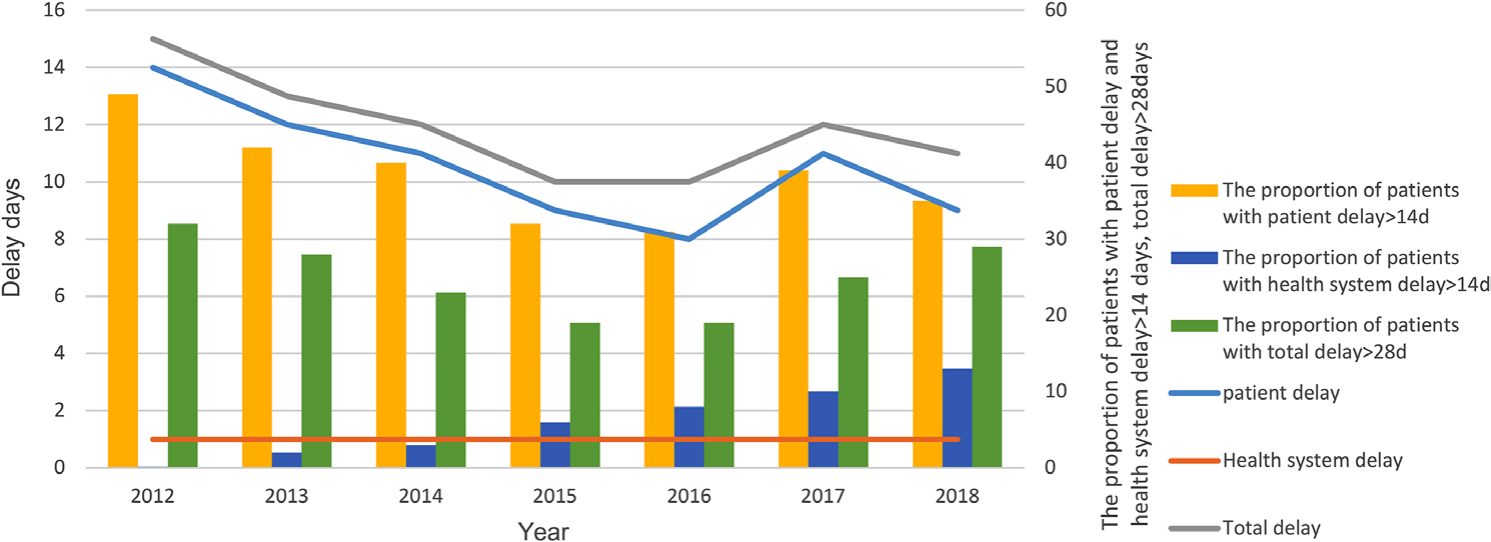
Patient delay, health system delay and total delay for TB patients from 2012 to 2018

Since 2012, median health system delay was maintained at 1 day. Median patient delay and median total delay showed an overall downward trend, with median patient delay decreasing from 14 days to 9 days and median total delay decreasing from 15 days to 11 days between 2012 and 2018. Both median patient delay and the median total delay showed a year-by-year downward trend since 2012 and reached a minimum in 2016 (8 days and 10 days). Median patient delay and median total delay increased again in 2017 but decreased in 2018. In addition, the proportion of patients who experienced patient delay > 14 days and that of total delay > 28 days showed an overall downward trend, decreasing from 49% to 35% and from 32% to 29%, respectively. However, between 2012 and 2018, the proportion of patients experiencing health system delay > 14 days showed a year-by-year upward trend from 0.2% to 13% (Table 2; Fig. 1).

**Table 2:** Patient delay, health system delay and total delay of TB patients from 2012 to 2018

	2012	2013	2014	2015	2016	2017	2018	*P*-value
	n=(650)	n=(607)	n=(528)	n=(564)	n=(502)	n=(479)	n=(440)	
**Patient delay (Median, IQR)**	14(5–32)	12(4–30)	11(4–25)	9(3–20)	8(3–19)	11(3–24)	9(3–25)	*P* < 0.001
**Patient delay > 14 d (n, %)**								*P* < 0.001
No	332(51)	353(58)	317(60)	382(68)	335(69)	292(61)	286(65)	
Yes	318(49)	254(42)	211(40)	182(32)	153(31)	187(39)	154(35)	
**Health system delay (Median, IQR)**	1(1–1)	1(1–1)	1(1–1)	1(1–1)	1(1–1)	1(1–1)	1(1–1)	*P* < 0.001
**Health system delay > 14 d (n, %)**								*P* < 0.001
No	649(99.8)	597(98)	514(97)	530(94)	447(92)	429(90)	382(87)	
Yes	1(0.2)	10(2)	14(3)	34(6)	41(8)	50(10)	58(13)	
**Total delay (Median, IQR)**	15(6–33)	13(5–32)	12(5–27)	10(4–22)	10(4–23)	12(4–30)	11(4–34)	*P* < 0.001
**Total delay > 28 d (n, %)**								*P* < 0.001
No	445(68)	435(72)	404(77)	459(81)	395(81)	358(75)	311(71)	
Yes	205(32)	172(28)	124(23)	105(19)	93(19)	121(25)	129(29)	

**Fig. 2:**
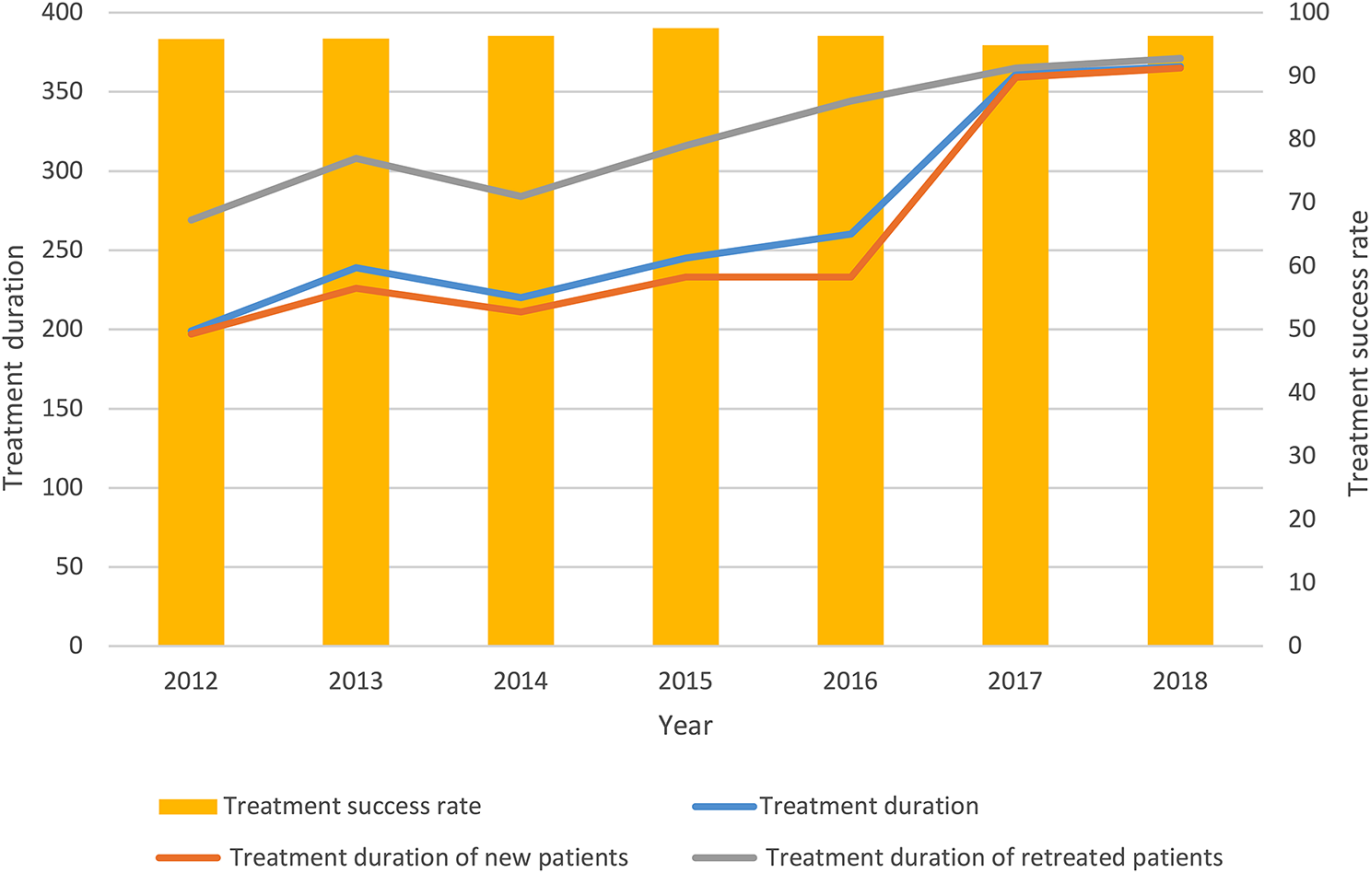
Treatment duration and treatment outcomes for TB patients from 2012 to 2018

Median treatment duration showed an upward trend from 2012 to 2016 and increased rapidly between 2017 and 2018. In addition, the treatment duration of new patients and that of retreated patients showed an overall upward trend from 2012 to 2018, increasing from 197 days to 365 days and from 269 days to 371 days, respectively. The integrated model achieved very high treatment success rates of above 90% from 2012 to 2018. The number of TB patients lost to follow-up was maintained at 0 between 2012 and 2013. However, this number showed an increase from 2014 to 2018 (Table 3; Fig. 2).

**Table 3:** Treatment duration and treatment outcomes of TB patients from 2012 to 2018

	2012	2013	2014	2015	2016	2017	2018	*P*-value
	n=(650)	n=(607)	n=(528)	n=(564)	n=(488)	n=(479)	n=(440)	
**Treatment duration (Median, IQR)**	199(192–229)	239(204–343)	220(195–329)	245(205–253)	260(196–363)	361(249–371)	366(305–377)	*P* < 0.001
New patient	197(191–209)	226(202–338)	211(193–317)	233(202–350)	233(193–361)	359(234–370)	365(292–377)	
Retreated patient	269(255–295)	308(225–364)	284(256–352)	316(266–356)	344(265–374)	365(347–373)	371(361–380)	
**Treatment outcomes (n, %)**								*P =* 0.305
Treatment success	623(95.8)	582(95.9)	508(96.3)	550(97.5)	470(96.3)	454(94.8)	428(96.3)	
Cured ^a^	196(30.1)	187(30.8)	160(30.3)	203(36)	130(26.6)	159(33.2)	160(36.4)	
Treatment completed ^b^	427(65.7)	395(65.1)	348(66.0)	347(61.5)	340(69.7)	295(61.6)	268(60.9)	
Unfavorable outcomes	27(4.2)	25(4.1)	20(3.7)	14(2.5)	18(3.7)	25(5.2)	12(2.7)	
Lost to follow-up ^c^	0(0)	0(0)	6(1.1)	5(0.9)	12(2.5)	15(3.1)	7(1.6)	
Treatment failed ^d^	22(3.4)	20(3.3)	8(1.5)	7(1.2)	3(0.6)	7(1.5)	5(1.1)	
Died ^e^	5(0.8)	5(0.8)	6(1.1)	2(0.4)	3(0.6)	3(0.6)	0(0)	

^a^ A smear positive patient who has completed the designated course of treatment and has two consecutive negative sputum test results, with one of them being a sputum test at the end of treatment

^b^ A smear negative patient who has completed the designated course of treatment and has a negative sputum test result at the end of treatment or no sputum test results; or a smear positive patient who has completed the designated course of treatment and has a negative sputum test result on the most recent occasion, with no sputum test results at the end of treatment

^c^ A patient whose treatment was interrupted for more than 2 consecutive months, or who is transferred out of the tuberculosis control agency without information or who have re-registered for treatment in another area within two months

^d^ A patient whose sputum smear is positive at 5 months or later during treatment. Before 2013, also included in this definition are patients found to harbour a multidrug-resistant (MDR) strain at any point of time during the treatment, as well as patients stopping treatment due to a serious adverse reaction to anti-tuberculosis medication. After 2014, patients found to harbour a multidrug resistance (MDR) were excluded and were not considered a treatment outcome

^e^ A patient who dies for any reason during the course of treatment

### Factors influencing diagnostic delay and treatment duration of TB patients in 2018

The univariate analysis suggested that patient delay > 14 days was significantly correlated with gender, *hukou* status and hospital level of initial diagnosis for TB (p < 0.05). Health system delay > 14 days was significantly correlated with age, *hukou* status, occupation, severity and hospital level of initial diagnosis for TB (p < 0.05). The total delay > 28 days was significantly correlated with gender, *hukou* status, severity and hospital level of initial diagnosis for TB (p < 0.05). Based on the multivariable regression analysis, female TB patients (OR = 0.569, 95% CI: 0.352–0.919) were less possible to have patient delay > 14 days, and TB patients who were initially diagnosed at the hospitals at prefectural level and above (OR = 2.459, 95% CI: 1.019–5.935) were more possible to have patient delay > 14 days. Migrant TB patients (OR = 4.578, 95% CI: 2.018–10.389) and TB patients who were initially diagnosed in hospitals at the prefectural level and above (OR = 7.305, 95% CI: 2.772–19.254) were more likely to experience total delay > 28 days, and female TB patients (OR = 0.464, 95% CI: 0.251–0.857) were less likely to have total delay > 28 days (Table 4).

**Table 4:** Factors influencing the probability of patient delay, health system delay (> 14 days) and total delay (> 28 days) for TB patients in 2018

Variables	Patient delay (n,%)			Health system delay (n,%)			Total delay (n,%)		
	≤14days	> 14days ^u^	Adjusted OR(95% CI)^m^	≤14days	> 14days ^u^	Adjusted OR(95% CI)^m^	≤28days	> 28days ^u^	Adjusted OR(95% CI) ^m^
Age>60y									
No	214(75)	123(80)		286(75)	51(88)		231(74)	106(82)	
Yes	72(25)	31(20)		96(25)	7(12)*	0.723 (0.182 to 2.879)	80(26)	23(18)	0.946(0.505 to 1.773)
**Gender**									
Male	194(68)	120(78)		269(70)	45(78)		213(69)	101(78)	
Female	92(32)	34(22)*	0.569(0.352 to 0.919)*	113(30)	13(22)		98(31)	28(22)*	0.464(0.251 to 0.857)*
***Hukou*** **status**									
Local	244(85)	92(60)		333(87)	3(5)		284(91)	52(40)	
Migrant	42(15)	62(40)***	2.098(0.950 to 4.633)	49(13)	55(95)***	-	27(9)	77(60)***	4.578 (2.018 to 10.389)***
**Occupation**									
Farmer	141(49)	76(49)		208(55)	9(16)		162(52)	55(43)	
Others	145(51)	78(51)		174(45)	49(84)***	2.494 (0.841 to 7.394)	149(48)	74(57)	
**Smear sputum results**									
Negativity	178(62)	93(60)		230(60)	41(71)		189(61)	82(64)	
Positivity	108(38)	61(40)		152(40)	17(29)	1.307 (0.432 to 3.947)	122(39)	47(36)	
**Treatment classification**									
New patients	254(89)	132(86)		334(87)	52(90)		276(89)	110(85)	
Retreated patient	32(11)	22(14)		48(13)	6(10)		35(11)	19(15)	
**Severe cases**									
No	190(66)	116(75)		254(67)	52(90)		203(65)	103(80)	
Yes	96(34)	38(25)	0.801(0.500 to 1.284)	128(33)	6(10)***	1.147(0.250 to 5.272)	108(35)	26(20)**	0785(0.441 to- 1.398)
**Cavity**									
No	180(63)	104(68)		244(64)	40(69)		194(62)	90(70)	
Yes	106(37)	50(32)		138(36)	18(31)		117(38)	39(30)	0.926(0.318 to 2.692)
**Hospital level of initial diagnosis for TB**									
County	259(91)	103(67)		360(94)	2(3)		299(96)	63(49)	
Prefectural and above	27(9)	51(33)***	2.459(1.019 to 5.935)***	22(6)	56(97)***	-	12(4)	66(51)***	7.305(2.772 to 19.254)***

***P < 0.001, **p < 0.01, *p < 0.05

^**u**^ The p value of univariate analysis is marked in this column with *

^**m**^ This column shows the confidence interval and p-value of multivariate analysis

^**g**^ There are too few samples in the control group, and the OR value is very large, so it is expressed with “-”

In our univariate analysis, age, hukou status, occupation and hospital level of initial diagnosis for TB were significantly correlated with the treatment duration of new patients (p < 0.05). Linear regression analysis confirmed that new TB patients>60 years (OR = 21.890, 95% CI: 2.731–41.048) tended to have longer treatment duration (Table 5). Our univariate analysis showed that *hukou* status and hospital level of initial diagnosis for TB were significantly correlated with the treatment duration of retreated patients (p < 0.05). Linear regression analysis confirmed that retreated TB patients who were initially diagnosed in hospitals at the prefectural and above-level (OR=-72.076, 95% CI: -110.932 - -33.220) tended to have shorter treatment duration (Table 6).

**Table 5:** Factors influencing the treatment duration of new TB patients in 2018

Variables	Treatment duration(Median, IQR)				
	Days	p-value ^u^	Adjusted β	95% CI	p-value
**Age>60y**					
No	364(247–376)				
Yes	370(360–379)	0.002	21.890	2.731 to 41.048	0.025
**Gender**					
Male	365(304–376)				
Female	367(246–379)	0.976			
***Hukou *** **status**					
Local	369(326–378)				
Migrant	345(227–364)	< 0.001	-1.068	-32.246 to 30.109	0.946
**Occupation**					
Farmer	368(348–378)				
Others	360(233–375)	0.002	-13.872	-30.127 to 2.383	0.094
**Smear sputum results**					
Negative	366(305–376)				
Positive	365(244–377)	0.508			
**Severe cases**					
No	366(331–376)				
Yes	365(215–377)	0.436			
**Cavity**					
No	366(310–366)				
Yes	365(236–377)	0.312			
**Hospital level of initial diagnosis for TB**					
County	369(309–378)				
Prefectural and above	341(244–356)	< 0.001	-19.195	-39.541 to -1.152	0.064

^**u**^*P*-value of univariate analysis

**Table 6:** Factors influencing the treatment duration of retreated TB patients in 2018

Variables	Treatment duration(Median, IQR)				
	Days	p-value ^u^	Adjusted β	95% CI	p-value
**Age>60y**					
No	367(356–379)				
Yes	374(365–382)	0.198	-17.802	-44.274 to 8.670	0.183
**Gender**					
Male	371(364–381)				
Female	367(307–374)	0.231			
***Hukou *** **status**					
Local	371(364–381)				
Migrant	354(277–370)	0.015	-35.363	-99.463 to 28.7	0.273
**Occupation**					
Farmer	371(362–381)				
Others	366(354–376)	0.463			
**Smear sputum results**					
Negative	367(359–377)				
Positive	372(364–382)	0.180	24.108	-0.941 to 49.157	0.059
**Severe cases**					
No	371(360–380)				
Yes	370(361–381)	0.719			
**Cavity**					
No	371(359–380)				
Yes	370(362–380)	0.874			
**Hospital level of initial diagnosis for TB**					
County	371(363–381)				
Prefectural and above	354(220–366)	0.015	-72.076	-110.932 to -33.220	0.003

^**u**^*P*-value of univariate analysis

## Discussion

### Summary of findings

In summary, median patient delay and median total delay showed an overall downward trend from 2012 since the implementation of the integrated model until 2018. Median health system delay was maintained at 1 day. The proportion of patients who experienced patient delay > 14 days and that of total delay > 28 days showed an overall downward trend while the proportion of patients experiencing health system delay > 14 days showed an upward trend from 2012 to 2018. Median treatment duration and the number of TB patients lost to follow-up both showed an overall upward trend from 2012 to 2018. Migrant TB patients and TB patients who were initially diagnosed in hospitals at the prefectural level and above were more likely to experience total delay > 28 days. Retreated TB patients who were initially diagnosed in hospitals at the prefectural level and above tended to have shorter treatment duration and new TB patients>60 years tended to have longer treatment duration.

### Comparison with literature

A relatively higher proportion (18-39%) of severe cases were treated in the integrated model since its implementation, underlying the policy’s original assumption that general hospitals have a stronger capacity to treat severe patients. However, this also indicates early detection and treatment of TB as challenge for the integrated model, although further research is needed to understand the impact of the integrated model on TB case management. In line with other studies [4, 7, 10], our study found that TB patients in the integrated model were associated with short median health system delay (1 days). This reflects the strength of the integrated model, which enables quicker diagnosis in or referral to the designated hospitals, especially for those patients who would usually visit general hospitals at the onset of symptoms [21]. However, our study reported that the probability of health system delay > 14 days showed an upward trend within the integrated model from 2012 to 2018. While further research is needed to explain this trend, this may be due to the relatively higher proportion of the severe patients and retreated patients and those first diagnosed in the hospitals at the prefectural level or above during the study period. In China, when a general hospital, either designated or not (including those at the prefectural level or above), receives and admits a severe patient, the patient may be hospitalized for some time as the attending doctor may need to do the diagnostic trial to confirm TB, or motivated by profit, they may retain presumptive TB patients for inpatient treatment before they are diagnosed.

Consistent with other studies [7, 16] our study found that TB patients treated in the integrated model had median patient delay decreasing from 14 to 9 days. Inconsistent with a previous study that showed a median total delay decreasing from 60 to 33 days [22], we found that TB patients in the integrated model had median total delay decreasing from 15 to 11 days. Median patient delay and median total delay showed an overall downward trend in the integrated model respectively, and we found a similar pattern for the proportion of patients who had patient delays > 14 days and total delays > 28 days. The literature has identified a number of socio-economic factors influencing patient delays, such as education, residency, health insurance and knowledge about TB [18, 23, 24], and our study may indicate the improved dissemination of knowledge about TB through the integrated model.

Few studies have assessed the treatment duration of TB patients within integrated models. Consistent with previous studies in China [13], our study found that the median treatment duration through the integrated model significantly exceeded the recommended days. The reasons for this excessive treatment duration need further investigation; however, there are a number of plausible explanations. According to the current treatment strategy, cessation of medication is mainly based on the sputum test results. For instance, a newly treated smear-positive patient should stop taking medication if they have negative sputum test results for two consecutive months at the end of the treatment course [19]. However, some doctors may recommend that patients take medication until their lesion stabilizes based on chest X-ray examinations. On the other hand, TB patients may concern about the potential relapse of TB and so ask for extended treatment [13]. Prolonged or overtreatment may be more common when TB service is integrated in the general hospitals where clinical medical culture prevails. Moreover, TB doctors may also struggle between their ‘mindlines’ and guidelines in providing ‘effective’ treatment for TB patients [25], since it is common for doctors to provide treatment based on their tactic knowledge, experiences and to be influenced by their communities of practices. Other plausible reasons could include the CDC’s ineffective supervision and lack of technical support for general hospitals, and perverse incentives for general hospitals that may encourage TB doctors to overprescribe unnecessary services [3, 26, 27] and prolong treatment duration due to the lack of goverement funding to support their essential infrastructure, equipment and other routine costs [28].

The integrated model achieved very high treatment success rates from 2012 to 2018. However, our study found that the number of TB patients lost to follow-up showed an overall upward trend from 2012 to 2018. This may indicate a weakness in the capability of the integrated model in case management and tracking. Moreover, the use of smear status as the main criterion for treatment responses may not be sufficient nor sensitive enough to measure treatment outcomes. It is therefore important to conduct systematic culturing in order to obtain a more accurate measurement of treatment outcomes.

Previous studies have found that older age contributes to health system delay [29]. Inconsistent with previous studies [30, 31], our univariate analysis showed that patients older than 60 were less likely to experience health system delay > 14 days. In contrast with previous studies [18, 31], our study presented a lower proportion of patients experiencing patient delay > 14 days and total delay > 28 days among the female patients. One plausible explanation is that men may bear a heavier economic burden as the main earner in a family, making it more difficult for them to sacrifice work time to seek medical assistance. In addition, our study found that TB patients with less severe infections were more likely to have instances of health system delay > 14 days and total delay > 28 days. Non-severe cases may experience mild symptoms of TB and be asymptomatic [16], making it difficult to be detected in the routine care, especially when the awareness amongst non-TB specialist healthcare providers is poor.

Consistent with other studies on migrant TB patient’s experiences of delays [32–35], our study found that migrant patients were more likely to encounter patient delay > 14 days and total delay > 28 days. This may be attributable to poor health awareness and health behaviors amongst migrant patients associated with their engagement with more precarious social and economic conditions, such as unstable work, low salary, poor living environment and poor working conditions [36–39]. This problem may also be explained by the fact that migrants are mostly uninsured in their host city and may therefore be less likely to visit health facilities at the onset of symptoms. In China, as national policies are mainly implemented based on the *hukou* system and transferring one’s *hukou* is exceedingly difficult, migrants largely do not have access to medical insurances or financial support in their host city and experience higher financial burden where they work and live than local *hukou* residents [36]. In addition, our univariate analysis demonstrated a higher risk of health system delay > 14 days amongst the migrant patients. As migrants tend to be younger [14, 39] and may have lighter symptoms, non-TB doctors may find it more difficult to recognize and identifiy infection. On the other hand, migrants may be concerned that they will lose their employment if they are diagnosed with TB [40] and so may delay in seeking out treatment.

Resembling a previous study in China [3], our study found that TB patients who were initially diagnosed at the hospitals at the prefectural level and above tended to have higher possibility of health system delay > 14 days. While our study found that the proportion of this patient group showed an upward trend, this coincides with the increasing number of migrant cases, with migrants prefering to seek medical treatment at prefectural-level hospitals [14] and tending to have longer health system delay > 14 days, as shown in our study. Furthermore, we found that TB patients who were initially diagnosed in hospitals at the prefectural level and above were more likely to have a higher risk of having patient delay > 14 days. This might be due to the fact that most of the patients visiting a hospital at prefectural level or higher are migrant TB patients who experienced longer patient delay [14].

Our univariate analysis showed that migrant patients, both new patients and retreated, were associated with shorter treatment duration. This may be due to migrant patients generally having lower income, higher mobility and a lower probability of completing treatment [36–39]. Our univariate analysis showed that new patients who were non-farmers were associated with shorter treatment duration. One possible explanation is that non-farmer patients may have less free working hours compared with farmer patients, which may result in poorer treatment compliance. Our study also showed that new TB patients>60 years were associated with longer treatment duration. This may be due to patients>60 years usually having more severe symptoms [31] and requiring longer treatment time. Finally, in our study, we found that new and retreated TB patients initially diagnosed in hospitals at the prefectural level and above tended to have shorter treatment duration. One possible reason is that most of the patients visiting at a higher-level hospital (i.e. prefectural-level hospital) tended to be migrant TB patients [14] who had higher mobility and faced more challenges in following treatment in a designated hospital.

### Policy implications

Despite the achievement of the integrated service delivery reform, a number of lessons can be generated to improve the integrated model of TB service delivery in China and in other similar contexts.

Firstly, given the significantly extended treatment duration and a tendency for more serious loss to follow-up in the integrated model, it is important for the health authority and CDC to strengthen the supervision and management of the clinical behavior in designated hospitals. Concurrently, the criteria of completing treatment in the designated hospitals should also be improved. It is necessary to strengthen the training for TB doctors, to improve their awareness of implementing evidence-based medicine in order to correct the deviation from the TB treatment protocol and find a balance between ensuring quality of care and compliance with TB guidelines. It is further necessary to improve financial support for designated hospitals to avoid potential profit-seeking behaviors.

Secondly, given the serious patient delay and total delay among migrant TB patients, it is necessary to enhance health education and promotion, increase medical insurance coverage among migrants, and improve the inter-regional transfer of health insurance. Furthermore, it is necessary to provide migrants with the necessary legal support to protect them from being made redundant after a confirmed diagnosis and with essential financial incentives to support their treatment [40, 41]. However, local residents may also have poor awareness of TB and it is necessary to strengthen public health promotion of TB and encourage people to seek out medical assistance and treatment at the onset of symptoms [42]. With the percentage of retreated patients identified annually remaininged high (> 10%), this further indicates the importance of strengthening the education and awareness for patients who had been infected with TB before to seek out care in time and improve early detection and treatment of TB.

Thirdly, our study suggests the potential of the integrated model in early detection and diagnosis of TB. However, this potential is contingent on the timely and accurate assessment of symptoms, especially as our study found that the probability of health system delay > 14 days increased in the integrated model during the study period. It is important to enhance the training for non-TB doctors on the identification of TB and timely referral of presumptive patients [42], especially those presenting with reinfection. It is important to improve early detection and address health system delay, especially amongst migrant, younger and non-severe patients who had higher risk of health systems delay > 14 days as per our study.

Finally, standardised TB care could be further integrated with or improved in other non-designated general hospitals including those hospitals at the prefecture level or above that have the capacity to diagnose and clinically manage TB cases. This would not only reduce the possibility of health system delay for TB patients but also improve the implementation of outpatient-based DOTS strategy.

### Limitations

Several limitations should be noted. First, our study may have limited generalizability as it is a case study. However, our study remains valuable as it reports the trend of diagnostic delays, treatment duration and outcomes over seven years and provides a detailed analysis of the factors influencing the delay and treatment duration for TB patients in the integrated model. Second, due to the data availability, we fail to compare the integrated model with the dispensary model, which limits our ability to explain the effectiveness of the integrated model. Third, we found a downward trend of diagnostic delay since the implementation of the integrated model and although we cannot exclude the impact of time on these changes, this at least suggests the integrated model could benefit the early TB detection and diagnosis. Fourth, due to the limitations of the routine electronic data, we failed to include more socio-demographic factors such as income and education, which could also influence the diagnostic delay [43–45]. Similarly, we failed to include non-patient related factors associated with treatment duration that may be important for explaining the prolonged treatment duration of the TB patients; in this study, we focused on the patient-related characteristics of prolonged treatment.

## Conclusions

The downward trend of diagnostic delay since the implementation of the integrated model in Cangnan County, Zhejiang may suggest the the potential of the integrated model in early detection and diagnosis of TB. However, concerns remain regarding the increasing proportion of health system delays > 14 days, increasing trend of treatment duration, and loss to follow-up in the integrated model during the study period. Our study suggests the importance of strengthening supervision and management of designated hospitals to optimize the treatment duration and improve retention of patients in TB care. Enhancing health education amongst TB patients, especially for migrant patients and improving training on TB identification and referral for non-TB doctors are also key for early TB detection and diagnosis in the integrated model.

## Data Availability

The datasets generated and/or analysed during the current study are not publicly available as TB Management Information System (TBIMS) is not an open access but an internal and administrative database for TB management in China, but are available from the corresponding author on reasonable request.
